# Effects of Ginsenoside Rg3 on Inhibiting Differentiation, Adipogenesis, and ER Stress-Mediated Cell Death in Brown Adipocytes

**DOI:** 10.1155/2021/6668665

**Published:** 2021-03-16

**Authors:** Seung-Nam Kim, Dae Hee Kim, Hyuek Jong Lee, Joon Seo Lim, Ju-Hee Lee, Sung Yun Park, Young Jun Koh

**Affiliations:** ^1^College of Korean Medicine, Dongguk University, Goyang, Republic of Korea; ^2^Center for Vascular Research, Institute for Basic Science (IBS), Daejeon, Republic of Korea; ^3^Clinical Research Center, Asan Medical Center, Seoul, Republic of Korea

## Abstract

**Objectives:**

Ginsenoside Rg3 (Rg3), a main active component of *Panax ginseng*, has various therapeutic properties in literatures, and it has been studied for its potential use in obesity control due to its antiadipogenic effects in white adipocytes. However, little is known about its effects on brown adipocytes.

**Methods:**

The mechanisms through which Rg3 inhibits differentiation, adipogenesis, and ER stress-mediated cell death in mouse primary brown adipocytes (MPBAs) are explored.

**Results:**

Rg3 significantly induced cytotoxicity in differentiated MPBAs but not in undifferentiated MPBAs. Rg3 treatment downregulated the expression of differentiation and adipogenesis markers and the level of perilipin in MPBAs while upregulating the expression of lipolytic Kruppel-like factor genes. Rg3 also induced lipolysis and efflux of triglycerides from MPBAs and subsequently increased proinflammatory cytokine levels. Notably, Rg3 treatment resulted in elevation of ER stress and proapoptotic markers in MPBAs.

**Conclusions:**

Our results demonstrate that Rg3 is able to selectively exert cytotoxicity in differentiated MPBAs while leaving undifferentiated MPBAs intact, resulting in the induction of ER stress and subsequent cell death in MPBAs via regulation of various genes related to adipocyte differentiation, adipogenesis, lipolysis, and inflammation. These results indicate that further studies on the potential therapeutic applications of Rg3 are warranted.

## 1. Introduction

The endoplasmic-reticulum (ER) is an important intracellular organelle for signal transduction that functions by storing and modulating calcium signals and is involved in the regulation of cellular processes, including protein folding and synthesis of lipids and sterols [1–3]. Pathological intracellular conditions such as low nutrient levels, hypoxia, and acidic pH level induce ER stress, thereby perturbing cellular homeostasis triggered by excessive activation of the unfolded protein response (UPR) in the ER [1, 4]. ER stress pathways are involved in cellular dysfunction and cell death, which contribute to various diseases such as Alzheimer's disease, Parkinson's disease, macular degenerative disease, inflammatory bowel disease, multiple sclerosis, rheumatoid arthritis, cancer, and type 2 diabetes [5].

Natural compounds have an abundance of structural and chemical diversity and are thus a major source of novel drugs for the prevention and treatment of various diseases [6]. Recent evidence has shown that while some natural compounds effectively reduce ER stress [7], others can be used to induce cellular death by enhancing ER stress [4]. Specifically, natural compounds, including paclitaxel and doxorubicin, have been frequently used as therapeutic regimens that function by inducing ER stress in malignant cells such as lung, breast, colorectal, gastric, prostate, and liver cancer cells [4, 6].

Ginsenoside Rg3 (Rg3), which is a major bioactive component of Korean red ginseng (*Panax ginseng*) [8], possesses various therapeutic benefits such as anticancer effects against various malignant cells [9], inhibition of platelet aggregation [10], antidepressant effects mediated via regulation of neurotransmitters [11], reduction of proinflammatory cytokines [12], and modulation of vascular functions [13]. Rg3 has also been shown to inhibit cell differentiation and viability of 3T3-L1 preadipocytes and reduce expression of adipogenic markers such as peroxisome proliferator-activated receptor gamma (PPAR*γ*) and CCAAT/enhancer binding protein alpha (C/EBP*α*) in livers of obese mice [14].

Brown adipocytes are thermogenic cells that generate heat in response to the sympathetic nerve system via a process known as nonshivering thermogenesis [15]. Interest in brown adipocytes has increased due to their potential therapeutic relevance for controlling pathological metabolic conditions such as obesity and diabetes, and a variety of molecular mechanisms underlying the differentiation of brown adipocytes have been described [16–18]. During differentiation of brown adipocytes, expression levels of thermogenic (e.g., uncoupling protein 1 (UCP1)) and adipogenic genes (e.g., PPAR*γ* and C/EBP*α*) are upregulated and consequently maintain the differentiated states of adipocytes [15]. Recent studies have also described many different molecular pathways, genetic alterations, and small molecules that modulate brown adipocyte differentiation [16–18].

In this study, we explored the mechanisms through which Rg3 inhibits differentiation, adipogenesis, and ER stress-mediated cell death in mouse primary brown adipocytes (MPBAs).

## 2. Methods

### 2.1. Reagents

Rg3, insulin, dexamethasone, 3-isobutyl-1-methylxanthine (IBMX), indomethacin, and 3,3′,5-triiodothyronine (T3) were purchased from Sigma-Aldrich (St. Louis, MO, USA). A lipid (Oil-Red O) staining kit was purchased from BioVision (Mountain View, CA, USA). 3-(4,5-methylthiazol-2-yl)-2,5-diphenyltetrazolium bromide (MTT) solution was purchased from ThermoFisher Scientific (Waltham, MA, USA). A triglyceride (TG) assay kit was obtained from Abcam (Cambridge, UK). Antibodies against PPAR*γ*, perilipin, endoplasmic reticulum oxidoreductin 1 alpha (ERO1L), phosphorylated eukaryotic initiation factor 2 alpha (Phospho-eIF2*α*), phosphorylated Akt (Phospho-Akt), total Akt, cleaved caspase-3, procaspase-3, and *β*-actin were purchased from Cell Signaling Technology (Danvers, MA, USA). Horseradish peroxide-conjugated secondary antibodies were purchased from Santa Cruz Biotechnology (Dallas, TX, USA).

### 2.2. Mouse Primary Brown Preadipocyte (MPBPA) Cell Culture and Differentiation

The protocols for isolation of MPBPAs were approved by the Institutional Animal Care and Use Committee of Dongguk University (approval no. IACUC-2017-017-3). Neonatal C57BL/6 mice were obtained from DBL Co., Ltd. (Seoul, Korea). We removed and dissected interscapular brown fat from C57BL/6 mice on postnatal day two. Tissues were then mixed with 500 *μ*L of collagenase and vortexed. After digestion of the fat pads, the tissues were passed through 100 *μ*m filters and centrifuged. Cells were then plated in 12-well plates (Corning Inc.) in culture medium (Dulbecco's modified Eagle's medium (DMEM) supplemented with 10% fetal bovine serum (FBS) and antibiotics) at 37°C in a 5% CO_2_ incubator. For immortalization, cells were infected using retroviruses encoding SV 40 T antigen. After selection with 20 *μ*g/mL of puromycin (Sigma-Aldrich), cells were maintained in culture medium and used in further experiments. For differentiation into mature brown adipocytes, MPBPAs were cultured for two days with differentiation inducer (10 *μ*g/mL of insulin, 0.25 *μ*M dexamethasone, 0.5 mM IBMX, 250 nM indomethacin, and 1nM T3) and further maintained in DMEM containing 10% FBS for three days. After differentiation of MPBPAs into MPBAs, Rg3 was added at the indicated concentrations and incubated for an additional two days.

### 2.3. Cell Viability Assay

Cytotoxic effects of Rg3 on MPBPAs and MPBAs were evaluated by MTT assay. Cells were cultured for 24 h in 24-well plates (Corning Inc.) in DMEM containing 1% FBS and the indicated doses of Rg3. After removal of the culture medium, cells were incubated with 300 *μ*L of MTT (0.5 mg/mL) for 2 h at 37°C in a 5% CO_2_ incubator. Formazan crystals in viable cells were dissolved in 200 *μ*L of DMSO, and the absorbance in each well was measured at 570 nm using a SpectraMax ELISA reader (Molecular Devices, San Jose, CA, USA).

### 2.4. Quantitative Real-Time Polymerase Chain Reaction Analysis

Total RNA from MPBAs was isolated using a ReliaPrep^TM^ RNA Miniprep Kit (Promega, Madison, WI, USA) according to the manufacturer's instructions. cDNA was synthesized from total RNA using a high-capacity RNA-to-cDNA kit (Applied Biosystems, Foster City, CA, USA). Polymerase chain reaction (PCR) was performed using a LightCycler 96 Probes Master Green Mix and a LightCycler 96 system (Roche, Basel, Switzerland). The PCR conditions were as follows: predenaturation for 10 min at 95°C, followed by 30 cycles of denaturation for 10 sec at 95°C, and annealing/extension for 20 sec at 55°C. Data were analyzed using the ΔΔCt method for relative quantification. Expression of each gene was normalized to 18S ribosomal RNA (18S rRNA) and expressed as fold changes relative to that of the DMSO control group. Primers for quantitative real-time PCR analysis are designed as followed: UCP1-F; GGCAAAAACAGAAGGATTGC, UCP1-R; TAAGCCGGCTGAGATCTTGT, PPAR*γ*-F; TCGCTGATGCACTGCCTATG, PPAR*γ*-R; GAGAGGTCCACAGAGCTGATT, C/EBP*α*-F; TCGGTGCGTCTAAGATGAGG, C/EBP*α*-R; TCAAGGCACATTTTTGCTCC, C/EBP*β*-F; TGACGCAACACACGTGTAACTG, C/EBP*β*-R; AACAACCCCGCAGGAACAT, KLF2-F; AATGACTCTGCCACCAGTTC, KLF2-R; GACCCGAGGGAAATAAGTCAAT, KLF3-F; CTACACAGGAAACGCCACAT, KLF3-R; GGAGAGAGAGAGAGAGAAAGAGAG, KLF5-F; CTGCCACTCTGCCAGTTAAT, KLF5-R; GAAGTGGATACGTCGCTTCTC, KLF7-F; CACAGGTGAGAAGCCTTACAA, KLF7-F; ACCTGTGTGTTTCCTGTAGTG, KLF16-F; GCCTCAGCAGGGATTTCTAT, KLF16-R; CAATGGCTTTCAGTGGAGTG, MCP1-F; GTCCCTGTCATGCTTCTGG, MCP1-R; GCTCTCCAGCCTACTCATTG.

### 2.5. Western Blot Analysis

Western blot analysis was performed as previously described [19]. Briefly, MPBAs were treated with the indicated doses of Rg3 for two days. Total proteins were extracted using lysis buffer containing protease and phosphatase inhibitors, followed by processing for SDS-PAGE. The blots were incubated with the following primary antibodies as appropriate: PPAR*γ*, perilipin, ERO1L, Phospho-eIF2*α*, CHOP, Phospho-Akt, total Akt, cleaved caspase3, total caspase3, and *β*-actin (all antibodies purchased from Cell Signaling Technology). An ECL Prime Western Blotting System (GE Healthcare) and ImageQuant LAS4000 (GE Healthcare) were used to detect protein bands. Densitometry analysis was performed using Image *J* software (NIH, Bethesda, MD, USA).

### 2.6. Oil-Red O Staining and Measurement of Lipid Contents

MPBAs were gently washed twice with PBS and then fixed with 10% formalin for 1 h at 37°C. After washing twice with distilled water (DW), the cells were stained with Oil-Red O solution (in 60% isopropanol) for 20 min and washed three times with DW. Each well of the culture plates was photographed with a Nikon Eclipse TS100 microscope (Nikon, Tokyo, Japan), and the tube areas were quantified using Image *J* software (NIH, Bethesda, MD, USA). For lipid quantification, Oil-Red O-stained MPBAs were dissolved in 1 mL of isopropanol for 1 h at room temperature (RT), and the absorbance in each well was measured at 495 nm using a SpectraMax ELISA reader (Molecular Devices, San Jose, CA, USA). Lipid contents were expressed as percentages relative to the DMSO control group.

### 2.7. Measurement of Total TG Contents

For total TG quantification, lipids were extracted according to the manufacturer's instructions. Briefly, MPBAs were homogenized in 1 mL of solution containing 5% NP-40 substitute (USB Corporation, Cleveland, OH, USA). The homogenates were then slowly heated to 80°C–100°C in a heating block for 2–5 min until the NP-40 became cloudy, followed by cooling to room temperature. Samples were subsequently centrifuged for 3 min to remove the insoluble materials. TG levels were measured by enzymatic assay (Abcam) and normalized to their respective protein concentrations.

### 2.8. Proteome Cytokines Profile Array Analysis

MPBAs were cultured for two days in DMEM containing 1% FBS with the indicated doses of Rg3. After collecting the media, inflammatory cytokines in the media were screened using a Mouse Proteome Cytokines Array kit panel A (R&D Systems, Minneapolis, MN, USA) according to the manufacturer's instructions. Signals were detected using an enhanced ECL Prime Western Blotting system (Sigma-Aldrich). Densitometry analysis was performed using Image *J* software (NIH).

### 2.9. Statistical Analysis

Statistical analyses were performed using SigmaPlot version 14.0 (Systat Software Inc., San Jose, CA, USA), and standard two-tailed Student's *t*-tests assuming unequal variances were used to identify differences between groups. *P* values <0.05 were considered statistically significant. Quantitative data are presented as the means ± SD.

## 3. Results

### 3.1. Rg3 Induces Greater Cytotoxicity in MPBAs than in MPBPAs

**Figure 1:**
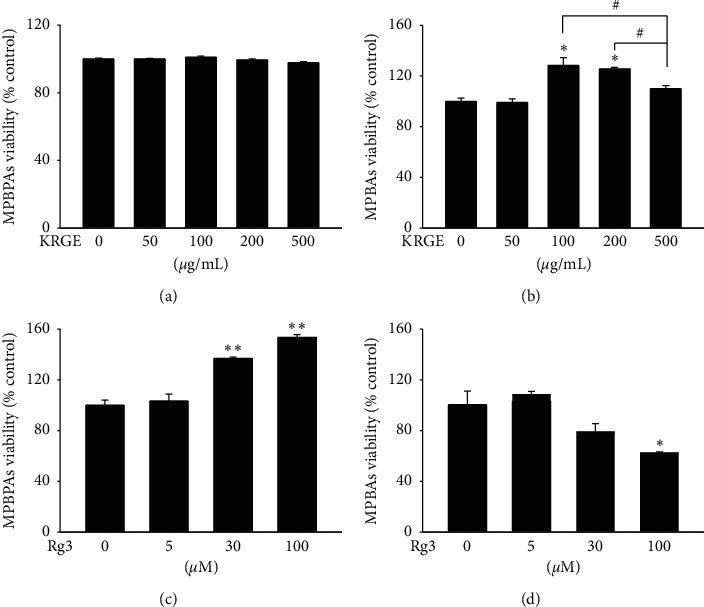
Effects of KRGE and Rg3 on cell viability of MPBPAs and MPBAs. (a–d) MPBPAs and MPBAs were treated with the indicated doses of KRGE and Rg3 for 72 h. The cytotoxicities of KRGE and Rg3 in MPBPAs and MPBAs were then measured by the MTT assay. The results are shown as percentages relative to the control. The graph represents the means ± SD (*n* = 4). ^*∗*^*P* < 0.01 and ^*∗∗*^*P* < 0.001 compared with the control. #*P* < 0.01 compared with 500 *μ*g/mL of KRGE treatment group.

To examine the cytotoxicity of Korean red ginseng extract (KRGE) and Rg3, MPBPAs and MPBAs were treated with KRGE and Rg3 at various dosages for 24 h, after which cell viability was evaluated by MTT assay. Significant cytotoxicity was not observed in MPBPAs when up to 500 *μ*g/mL of KRGE (Figure 1(a)) and up to 100 *μ*M Rg3 were applied (Figure 1(c)). In addition, KRGE induced cell proliferation of MPBAs when applied at doses of 100 and 200 *μ*g/mL, whereas 500 *μ*g/mL of KRGE treatment hindered cell proliferation (Figures 1(a) and 1(b)). Moreover, Rg3 but not KRGE enhanced cell proliferation in MPBPAs in a dose-dependent manner when applied at concentrations up to 100 *μ*M (Figure 1(c)). In contrast, Rg3 induced cytotoxicity in MPBAs when applied at 100 *μ*M (Figure 1(d)), which was the dosage that induced cell proliferation in MPBPAs (Figures 1(b)–1(d)).

### 3.2. Rg3 Inhibits Expression of Genes and Proteins Associated with Differentiation of MPBPAs into MPBAs

**Figure 2:**
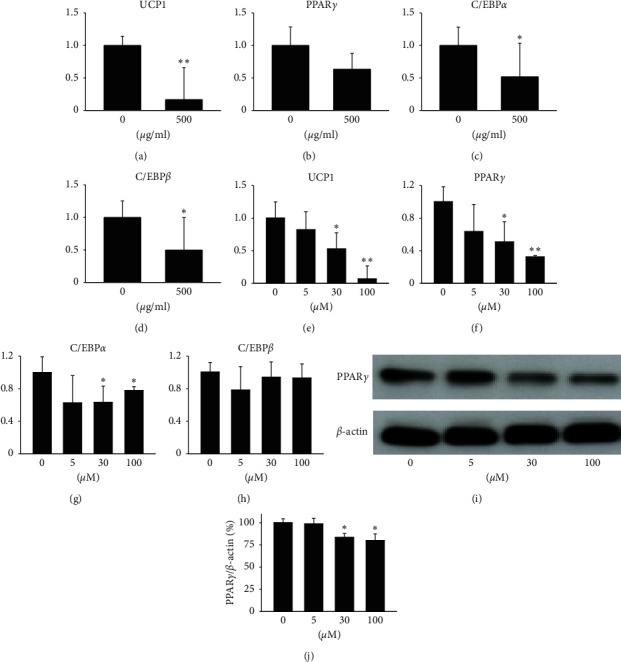
Inhibitory effects of KRGE and Rg3 on expression of genes and proteins associated with differentiation of MPBPAs into MPBAs. MPBAs were treated with the indicated doses of KRGE and Rg3 for 72 h. Effects of (a–d) KRGE and (e–h) Rg3 in MPBAs on gene expression of (a, e) UCP1, (b, f) PPAR*γ*, (c, g) C/EBP*α*, and (d, h) C/EBP*β* were measured by quantitative real-time PCR analysis. The results indicate fold increases relative to the control. The graph represents the means ± SD (*n* = 4). ^*∗*^*P* < 0.01 and ^*∗∗*^*P* < 0.001 compared with the control. (i) The effects of Rg3 in MPBAs on protein expression of PPAR*γ* were examined by Western blotting analysis. *β*-actin was used as a control. (j) Quantitative densitometric analysis of PPAR*γ*/*β*-actin. The results are shown as a ratio to the control. The graph represents the means ± SD (*n* = 4). ^*∗*^*P* < 0.01 and ^*∗∗*^*P* < 0.001 compared with the control.

To elucidate the inhibitory effects of KRGE and Rg3 on the differentiation of MPBPAs into MPBAs, MPBPAs were treated with KRGE (0 or 500 *μ*g/mL) and Rg3 (0–100 *μ*M) during differentiation, after which the expression of genes and proteins related to brown adipocyte differentiation was determined by quantitative real-time PCR and Western blotting analyses, respectively. At 500 *μ*g/mL, KRGE significantly downregulated mRNA levels of UCP1, C/EBP*α*, and C/EBP*β* by >50% compared with those of the control group (Figures 2(a)–2(d)). At 30 and 100 *μ*M, Rg3 downregulated mRNA levels of UCP1, PPAR*γ*, and C/EBP*α* by >50% compared with those of the control group as well (Figures 2(e)–2(h)). Furthermore, Rg3 at concentrations of 30 and 100 *μ*M reduced the protein level of PPAR*γ* by approximately 20% in MPBAs (Figures 2(i) and 2(j)).

### 3.3. Rg3 Induces Expression of Antiadipogenic KLFs Genes and Inhibits Expression of Lipid-Coated Proteins in MPBAs

**Figure 3:**
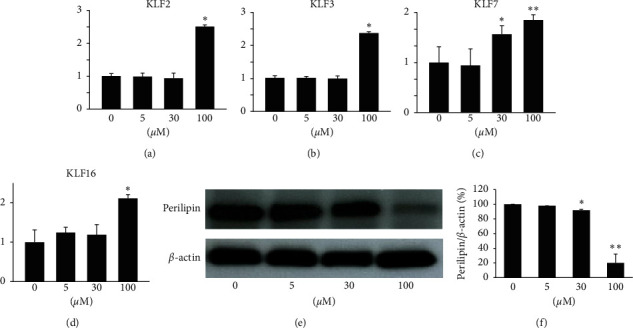
Lipolytic effects of Rg3 on gene and protein expression associated with differentiation of MPBPAs into MPBAs. MPBAs were treated with the indicated doses of Rg3 for 72 h. (a–d) Effects of Rg3 in MPBAs on expression levels of (a) KLF2, (b) KLF3, (c) KLF7, and (d) KLF16 were measured by quantitative real-time PCR analysis. The results indicate fold increases relative to the control. The graph represents the means ± SD (*n* = 4). ^*∗*^*P* < 0.01 and ^*∗∗*^*P* < 0.001 compared with the control. (e) The effects of Rg3 in MPBAs on protein expression of perilipin were examined by Western blotting analysis. *β*-actin was used as a control. (f) Quantitative densitometric analysis of perilipin. The results are shown as a ratio to the control. The graph represents the means ± SD (*n* = 4). ^*∗*^*P* < 0.01 and ^*∗∗*^*P* < 0.001 compared with the control.

Previous studies have shown that Kruppel-like factor (KLF) genes are associated with antilipogenesis. Specifically, KLF2 is a negative regulator of adipogenesis, KLF3 and KLF7 expression is reduced in differentiated adipocytes, and KLF16 is specifically expressed in brown adipocytes and has been reported to be downregulated during differentiation. As such, to investigate whether or not Rg3 inhibits the lipogenesis of MPBAs, we treated MPBPAs with Rg3 (0–100 *μ*M) during differentiation and then examined the expression of KLF genes by quantitative real-time PCR. Rg3 (100 *μ*M) significantly upregulated the expression of KLF2, KLF3, KLF7, and KLF6 in MPBAs (Figures 3(a)–3(d)). To further elucidate the lipolysis effects of Rg3 on differentiation of MPBPAs into MPBAs, cells were treated with Rg3 (0–100 *μ*M) during MPBPA differentiation, after which the expression of perilipin, a lipid-coated protein associated with lipid storage of adipocytes, was measured. Rg3 downregulated the protein expression of perilipin (Figures 3(e) and 3(f)), which is in line with its antiadipogenic effects in MPBAs (Figures 3(a)–3(f)). These results indicate that Rg3 inhibits adipogenesis and induces lipolysis in MPBAs.

### 3.4. Rg3 Induces Lipolysis in MPBAs

**Figure 4:**
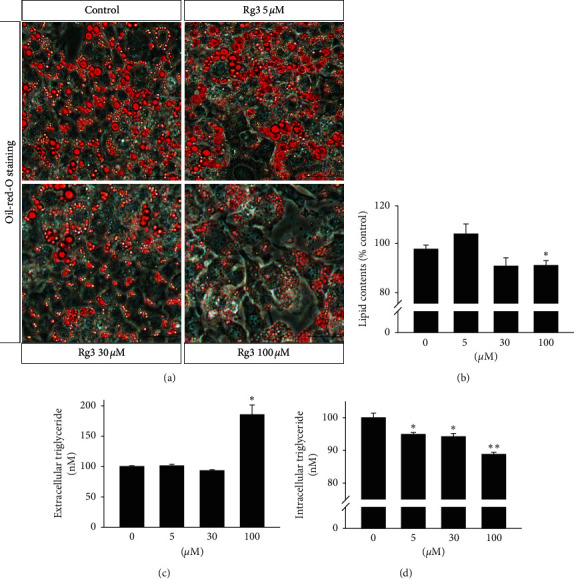
Effects of Rg3 on release of lipid contents from MPBAs. MPBAs were treated with the indicated doses of Rg3 for 72 h. (a) Representative images of Oil-Red O staining for lipid droplets (red) in MPBAs treated with Rg3 at the indicated doses. Bars, 50 *μ*m. (b) The effects of Rg3 in MPBAs on release of lipid contents were quantified by enzymatic assay. The graph shows the means ± SD (*n* = 4). ^*∗*^*P* < 0.01 compared with the control. (c and d) The effects of Rg3 in MPBAs on (c) extracellular and (d) intracellular TG release were quantified by enzymatic assay. The graph shows the means ± SD (*n* = 4). ^*∗*^*P* < 0.01 compared with the control.

To investigate whether or not Rg3 induces lipolysis in MPBAs, we treated MPBPAs with Rg3 (0–100 *μ*M) during differentiation, after which lipid contents and extracellular and intracellular TG levels were examined. First, Oil-Red O staining was performed to examine the extent of lipid accumulation in MPBAs, in either the presence or absence of Rg3. Representative images of Oil-Red O staining show that Rg3 (30 and 100 *μ*M) significantly suppressed lipid accumulation in MPBAs (Figure 4(a)). At 100 *μ*M, Rg3 significantly reduced lipid contents in MPBAs (Figure 4(b)). Additionally, Rg3 increased the amount of extracellular TG in MPBAs by approximately 70% compared with the control (Figure 4(c)). In addition, the intracellular TG content of MPBAs in the presence of Rg3 (5–100 *μ*M) was reduced by approximately 10% compared with the control (Figure 4(d)). These results show that Rg3 induces lipolysis in MPBAs.

### 3.5. Rg3 Induces Expression of Inflammatory Cytokines and Monocyte Chemoattractant Protein-1 (MCP-1) in MPBAs

**Figure 5:**
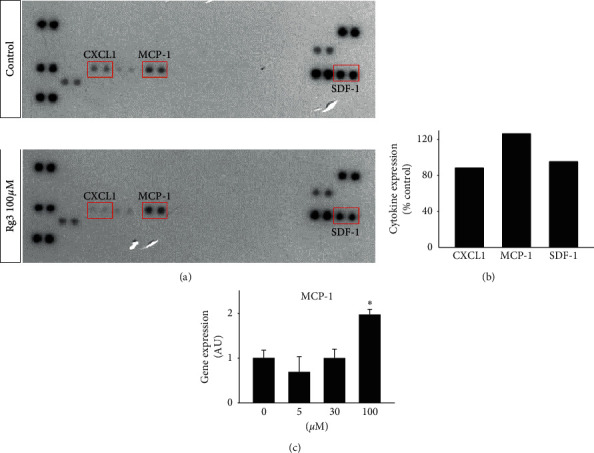
Effects of Rg3 on expression of inflammatory cytokines in MPBAs. MPBAs were treated with the indicated doses of Rg3 for 72 h. (a) The effects of Rg3 on MPBAs on expression of C-X-C motif chemokine ligand 1 (CXCL1), MCP-1, and stromal cell-derived factor 1 (SDF-1) were measured by Proteome Cytokines Profiler Array analysis. (b) Densitometric analysis of the Proteome Cytokines Profiler Array in (a). The results indicate fold increases relative to the control. The graph represents the means. (c) The effects of Rg3 in MPBAs on MCP-1 gene expression were examined by quantitative real-time PCR analysis. The graph shows the means ± SD (*n* = 4). ^*∗*^*P* < 0.01 compared with the control.

To determine whether or not Rg3 induces expression of inflammatory cytokines in MPBAs, we treated MPBPAs with Rg3 (0 and 100 *μ*M) during differentiation and then analyzed the samples using a Proteome Cytokines Profiler Array. Rg3 induced MCP-1 expression and inhibited CXCL-1 and SDF-1 expression in MPBAs (Figures 5(a) and 5(b)). We further investigated whether or not Rg3 induces MCP-1 expression in MPBAs. To accomplish this, we treated MPBPAs with Rg3 (0–100 *μ*M) during differentiation and then examined MCP-1 gene expression by quantitative real-time PCR. The results show that 100 *μ*M Rg3 significantly induced expression of MCP-1 in MPBAs (Figure 5(c)).

### 3.6. Rg3 Induces ER Stress and Subsequent Apoptosis Signaling of MPBAs

**Figure 6:**
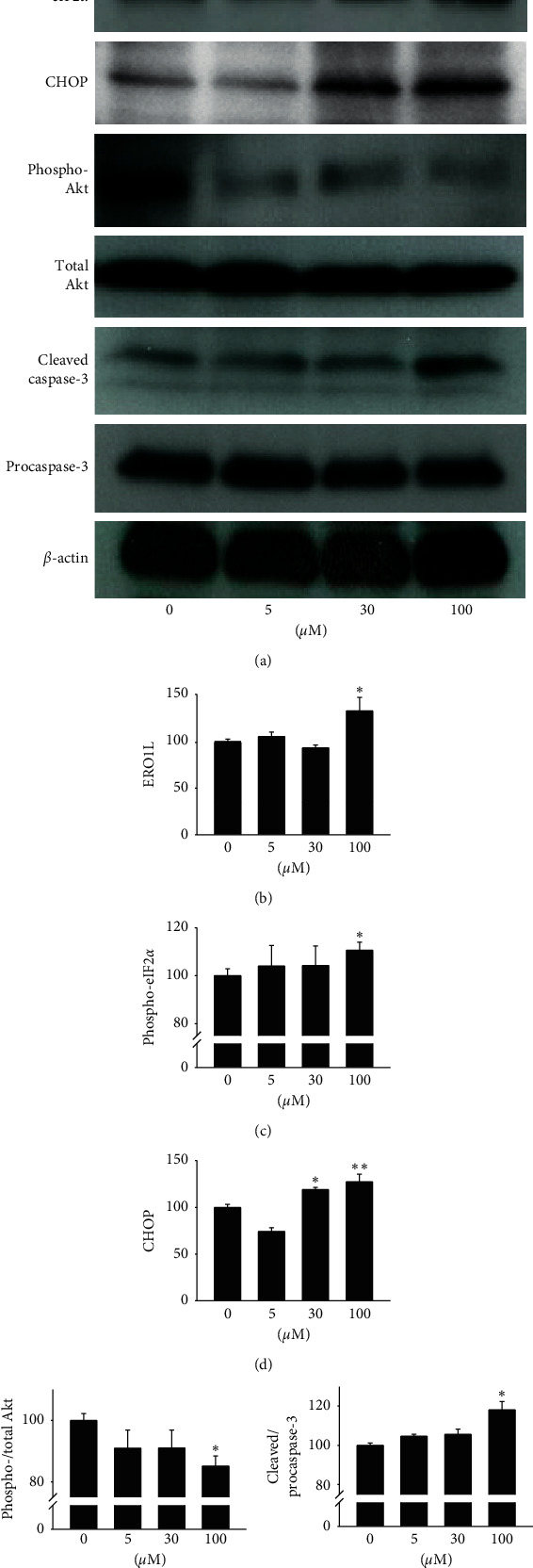
Effects of Rg3 on induction of ER stress and apoptosis signaling in MPBAs. MPBAs were treated with the indicated doses of Rg3 for 72 h. (a) The levels of ERO1L, phosphorylated eIF2*α* (Phospho-eIF2*α*), CHOP, phosphorylated Akt (Phospho-Akt), total Akt, cleaved caspase-3, procaspase-3, and *β*-actin were examined by Western blot analysis. (b–f) Quantitative densitometric analysis of (a). The results are shown as ratios to the control (total Akt, procaspase-3, and *β*-actin). The graph shows the means ± SD (*n* = 3). ^*∗*^*P* < 0.01 and ^*∗∗*^*P* < 0.001 compared with positive control.

To determine whether or not Rg3 induces ER stress and subsequent cell death signaling of MPBAs, we treated MPBPAs with Rg3 (0–100 *μ*M) during differentiation and then examined the expression levels of proteins associated with ER stress and cell death. Rg3 treatment induced expression of specific markers of the UPR pathway in response to ER stress, including ERO1L, Phospho-eIF2*α*, and CHOP (Figures 6(a)–6(d)). As a result, cleavage of Akt phosphorylation was reduced (Figures 6(a) and 6(e)), whereas cleavage of procaspase-3 increased (Figures 6(a) and 6(f)), leading to induction of apoptosis in MPBAs. These results show that Rg3 induces ER stress signals, resulting in functional impairment through apoptosis of MPBAs.

## 4. Discussion

Natural compounds represent an excellent source of novel drugs by providing diverse structural bases and chemical multiformity [6]. Recent evidence has shown that natural compound derivatives such as paclitaxel, doxorubicin, and artemisinin have therapeutic efficacies for various diseases, including tumors and malaria [4, 6, 20]. Rg3, an important natural compound in Korean red ginseng (*Panax ginseng*) [8], has demonstrated significant therapeutic effects, including inhibition of cancer progression, prevention of platelet aggregation, regulation of neurotransmitters, attenuation of inflammatory cytokines, and modulation of vascular tones [10–13]. Rg3 also has inhibitory effects on the adipogenic properties of mouse and human white preadipocytes [14], although little is known about its effects on brown preadipocytes or differentiated brown adipocytes. Therefore, we investigated the effects of Rg3 on brown adipocytes, which may be used to modulate metabolic characteristics.

Recent studies have characterized and distinguished the variable gene and phenotype expression between undifferentiated and differentiated cells, including white and brown adipocytes [21–24], which may be associated with differential sensitivity to stimuli such as small molecules under differentiation states [25, 26]. Differentiated cells are generally more sensitive to stimulation than undifferentiated cells. For example, huprines, which are inhibitors of acetylcholinesterases, show neuroprotective effects via cholinergic receptors only in differentiated pheochromocytoma [27]. Therefore, we speculate that KRGE and Rg3 may have distinct effects in undifferentiated (MPBPAs) and differentiated (MPBAs) brown adipocytes. We observed that KRGE increased proliferation of MPBAs at concentrations of 100 and 200 *μ*g/mL but inhibited proliferation at 500 *μ*g/mL (Figures 1(a) and 1(b)). Interestingly, Rg3, a main active component of KRGE, showed a significantly greater cytotoxicity in MPBAs than in MPBPAs (Figures 1(c) and 1(d)). Moreover, we observed that KRGE and Rg3 inhibited the expression of specific markers for mature brown adipocytes, such as UCP1, and differentiation markers of adipogenesis, including PPAR*γ*, C/EBP*α*, and C/EBP*β*, in MBPAs (Figures 2(a)–2(j)). These results show that Rg3 effectively inhibits cell differentiation by suppressing related genes in MPBAs.

KLFs, which are zinc finger proteins, regulate various mechanisms of energy homeostasis in many types of cells, including white and brown adipocytes [28]. Among KLF family genes, KLF2 [29], KLF3 [30], KLF7 [31], and KLF16 [32] are known to suppress adipocyte differentiation. KLF2, which is abundant in preadipocytes but not mature adipocytes, directly inhibits PPAR-*γ* promoter activity, and overexpression of KLF2 has been shown to inhibit PPAR*γ* and C/EBP*α* expression with no significant effect on C/EBP*β* and C/EBP*δ*, leading to overall negative regulation of adipogenesis [29]. KLF3 also inhibits adipocyte differentiation via suppression of C/EBP-*α* expression by binding to its promoter [30]. Similarly, KLF7 overexpression has been shown to downregulate the expression of PPAR*γ*, C/EBP*α*, adipocyte protein aP2, and adipsin in human preadipocytes, whereas C/EBP*β* and C/EBP*δ* expression are not altered [31]. Moreover, KLF16, which is downregulated during differentiation of brown adipocytes, has been identified as a transcription factor that inhibits adipogenesis of primary brown preadipocytes and expression of PPAR*γ* and aP2 [32]. Perilipin, a lipid-coated protein, inhibits lipolysis by acting as a physical barrier against lipase-induced hydrolysis of triacylglycerol [33]. Therefore, we examined whether or not Rg3 affects the expression of KLFs and perilipin. We observed that Rg3 increased the expression of KLF2, KLF3, KLF7, and KLF16 in MPBAs (Figures 3(a)–3(d)) and reduced the expression of perilipin (Figures 3(e) and 3(f)). As a result, Rg3 effectively inhibited the characteristic features of adipogenesis while inducing those of lipolysis, including reduction of lipid contents and elevation of TG secretion in MBPAs (Figures 4(a)–4(d)). Considering the effects of Rg3 on the expression of antiadipogenic and lipolysis-related markers of brown adipocytes, we suggest that Rg3 hinders adipogenesis in MPBPAs. In addition, a previous study showed that genetic silencing or deletion of perilipin in adipocytes and adipose tissues enhances lipolysis and consequently induces inflammation [14, 34]. Recent data also showed that inflammation could have inhibitory effects on UCP1 expression in brown adipose tissue by modulating sirtuin-1, which is rescued by resveratrol, a sirtuin-1 activator [35]. Consistently, our results show that Rg3 inhibited expression of UCP1 (Figure 2(e)) and induced upregulation of inflammatory cytokines such as MCP1 in MPBAs (Figures 5(a)–5(c)), and these changes were accompanied by a significant downregulation of perilipin expression (Figures 3(e) and 3(f)). These results show that Rg3 may induce inflammation by enhancing lipolysis in MPBAs.

Next, we further examined whether or not Rg3 induced ER stress in MBPAs, which led to inhibition of cell differentiation in MPBPAs and consequent cell death. Deng et al. previously showed that ER stress induced by thapsigargin, tunicamycin, and brefeldin A can lead to lipolysis via cyclic adenosine monophosphate/protein kinase A and ERK signaling in adipocytes [36]. This lipolytic activity caused by ER stress may contribute to lipotoxicity, which is associated with various metabolic diseases via induction of persistent efflux of free fatty acids from adipocytes [36]. Therefore, we explored whether or not Rg3 induced ER stress in MPBAs. We found that Rg3 significantly elevated the levels of ER stress-related markers in MBPAs, including ERO1L, phosphorylated eIF2*α*, and CHOP (Figure 6(a)–6(d)). Moreover, inactivation of Akt but not ERK has been found to induce CHOP expression and cause cell death [37]. Conversely, ER stress-induced apoptosis has been shown to be partially mediated by signaling through the Akt pathway [38]. These results collectively suggest that Akt plays a crucial role in ER-stressed states and is thus an important signaling pathway in ER stress-related disorders [37, 38]. Similarly, we demonstrated that Rg3 inhibited Akt phosphorylation in MBPAs (Figures 6(a) and 6(e)), suggesting that Rg3 induces ER stress in MPBAs. Severe ER stress can induce apoptotic cell death, which is initiated by phosphorylation of eIF2*α* [3, 39]. Moreover, upregulation of CHOP has been shown to result in induction of genes that hinder cell cycling, thereby inducing apoptosis [3]. Thus, an imbalanced cell response can induce cell death under states of ER stress [3, 39]. In addition, ER stress induces serum free fatty acid production, impairs glucose tolerance, and increases expression of CHOP and caspase-3 in adipose tissues, thereby leading to adipocyte apoptosis by elevating intracellular free fatty acid and calcium levels [40]. We also demonstrated that Rg3 induced cleavage of procaspase-3 in MBPAs, which may have led to apoptosis (Figures 6(a) and 6(f)).

## 5. Conclusion

In conclusion, we showed that Rg3, a major bioactive component of Korean red ginseng (*Panax ginseng*), sensitively induced cytotoxicity in differentiated brown adipocytes at dosages not cytotoxic to undifferentiated cells. Rg3 also inhibited differentiation and adipogenesis while increasing lipolysis and inflammation in differentiated brown adipocytes, thereby inducing ER stress and subsequent cell death. Considering that white and brown adipocytes constitute the two main axes of metabolic regulation in the human body, our results concerning the effects of Rg3 on brown adipocytes indicate that further studies investigating the therapeutic applications of Rg3 to obesity and related metabolic disorders are warranted.

## Data Availability

The data and materials used in the study are available upon request from the corresponding authors.
